# 3D black blood cardiovascular magnetic resonance atlases of congenital aortic arch anomalies and the normal fetal heart: application to automated multi-label segmentation

**DOI:** 10.1186/s12968-022-00902-z

**Published:** 2022-12-15

**Authors:** Alena U. Uus, Milou P. M. van Poppel, Johannes K. Steinweg, Irina Grigorescu, Paula Ramirez Gilliland, Thomas A. Roberts, Alexia Egloff Collado, Mary A. Rutherford, Joseph V. Hajnal, David F. A. Lloyd, Kuberan Pushparajah, Maria Deprez

**Affiliations:** 1 School of Imaging Sciences and Biomedical Engineering, King’s College London, London, UK; 2 Department of Congenital Heart Disease, Evelina London Children’s Hospital, London, UK; 3 Centre for the Developing Brain, King’s College London, London, UK; 4 Clinical Scientific Computing, Guy’s and St Thomas’ NHS Foundation Trust, London, UK

**Keywords:** Congenital aortic arch anomalies, 3D fetal MRI, Heart atlas, Vessel segmentation

## Abstract

**Background:**

Image-domain motion correction of black-blood contrast T2-weighted fetal cardiovascular magnetic resonance imaging (CMR) using slice-to-volume registration (SVR) provides high-resolution three-dimensional (3D) images of the fetal heart providing excellent 3D visualisation of vascular anomalies [1]. However, 3D segmentation of these datasets, important for both clinical reporting and the application of advanced analysis techniques is currently a time-consuming process requiring manual input with potential for inter-user variability.

**Methods:**

In this work, we present novel 3D fetal CMR population-averaged atlases of normal and abnormal fetal cardiovascular anatomy. The atlases are created using motion-corrected 3D reconstructed volumes of 86 third trimester fetuses (gestational age range 29-34 weeks) including: 28 healthy controls, 20 cases with postnatally confirmed neonatal coarctation of the aorta (CoA) and 38 vascular rings (21 right aortic arch (RAA), 17 double aortic arch (DAA)). We used only high image quality datasets with isolated anomalies and without any other deviations in the cardiovascular anatomy.In addition, we implemented and evaluated atlas-guided registration and deep learning (UNETR) methods for automated 3D multi-label segmentation of fetal cardiac vessels. We used images from CoA, RAA and DAA cohorts including: 42 cases for training (14 from each cohort), 3 for validation and 6 for testing. In addition, the potential limitations of the network were investigated on unseen datasets including 3 early gestational age (22 weeks) and 3 low SNR cases.

**Results:**

We created four atlases representing the average anatomy of the normal fetal heart, postnatally confirmed neonatal CoA, RAA and DAA. Visual inspection was undertaken to verify expected anatomy per subgroup. The results of the multi-label cardiac vessel UNETR segmentation showed 100$\%$ per-vessel detection rate for both normal and abnormal aortic arch anatomy.

**Conclusions:**

This work introduces the first set of 3D black-blood T2-weighted CMR atlases of normal and abnormal fetal cardiovascular anatomy including detailed segmentation of the major cardiovascular structures. Additionally, we demonstrated the general feasibility of using deep learning for multi-label vessel segmentation of 3D fetal CMR images.

**Supplementary Information:**

The online version contains supplementary material available at 10.1186/s12968-022-00902-z.

## Background

The black-blood contrast in T2-weighted single shot turbo spin echo (SSTSE) sequence widely used in fetal cardiovascular magnetic resonance (CMR) provides useful visualisation of fetal cardiovascular anomalies [2]. However, 2D-only slice-wise analysis is affected by uncontrolled fetal motion during image acquisition, affecting visualisation of small and complex 3D cardiovascular structures. Application of novel slice-to-volume registration (SVR) motion correction tools [3–5] allow reconstruction of motion-corrupted 2D datasets. The resulting high-resolution isotropic 3D images of the fetal thorax demonstrate excellent 3D visualisation of the fetal extracardiac vasculature providing adjunct diagnostic information to fetal echocardiography [1].

At our institution, main clinical referral indications for 3D T2-weighted fetal CMR [1] include suspected coarctation of the aorta (CoA) and vascular rings i.e. right aortic arch (RAA) with aberrant left subclavian artery (ALSA) and double aortic arch (DAA). However, despite the reported strong diagnostic performance of 3D fetal CMR [1, 6], there is a lack of formalisation of the CMR appearance of fetal cardiovascular anatomy. 3D segmentation of the fetal heart from motion-corrected volumes [1] is valuable for clinical reporting pipelines and application of advanced image analysis (i.e. statistical shape analysis). Commonly, 3D segmentation in CMR in general is useful for 3D visualisation and clinical reporting as well as advanced image analysis methods. However, the current 3D segmentation approach for black blood fetal CMR [1] relies of semi-automatic parcellation based on thresholding. It may not clearly differentiate between adjacent vessels and requires time-consuming manual refinement, which is vulnerable to inter-observer variability. The time required for refinement further increases in case of multiple labels.

### Related work

While the majority of the reported fetal CMR image processing methods have been focused only on the brain region, their application is being gradually extended to the rest of the fetal body. For instance, the existing various implementations of 3D SVR reconstruction methods [3, 4, 7] already showed to improve information content of CMR for the fetal heart [1] as well as the other body organs [8]. In addition to visual inspection, the reconstructed 3D CMR images are also used for volume rendering as well as biometry [6] and segmentation for quantitative analysis [9].

The results of the recent fetal brain MRI segmentation challenge [10] demonstrated that the modern automated segmentation solutions based on deep learning show promising results for both normal and abnormal fetal brain anatomy. However, so far, the reported methods were limited to the brain region only and used either the UNet-based solutions with training on manual labels [11, 12] or classical label propagation approaches that notably required significant amount of manual editing [13, 14].

Development of new segmentation methods for specific anatomy structures (e.g., the fetal heart) and MRI acquisition protocols would require definition of a reference parcellation protocol, which is conventionally performed in the atlas space. In this case, an atlas is a 3D image representing population-averaged anatomy that is generated by averaging co-aligned 3D MRI images from specific cohorts. The existing examples include spatio-temporal 3D MRI atlases of the normal fetal brain at different gestational ages [15] or specific brain anomalies [16]. However, so far, there have been no reported atlases of the fetal body.

### Contributions

In this work, we present the first 3D fetal CMR atlases of normal fetal cardiovascular anatomy and three subgroups of vascular anomalies (CoA, RAA with ALSA and DAA) generated from 87 fetal CMR datasets including formalised multi-label parcellations of the major cardiovascular structures. This contributes to the automation of 3D SVR-based reporting pipelines and quantitative analysis. We also perform evaluation of the feasibility of atlas-guided registration and deep learning methods for automated 3D multi-label vessel segmentation. In addition, the potential limitations of the network were investigated on unseen datasets including 3 early gestational age (22 weeks) and 3 low signal-to-noise (SNR) cases (Appendix).

## Methods

### Datasets and preprocessing

We included 92 fetal CMR datasets scanned at Evelina London Children’s Hospital/ St Thomas’ Hospital, London (UK). Data use for this project was subject to written informed consent of the participants (REC: 14/LO/1806 (iFIND-2 project) and REC 07/H0707/105).[17]

**Fig. 1:**
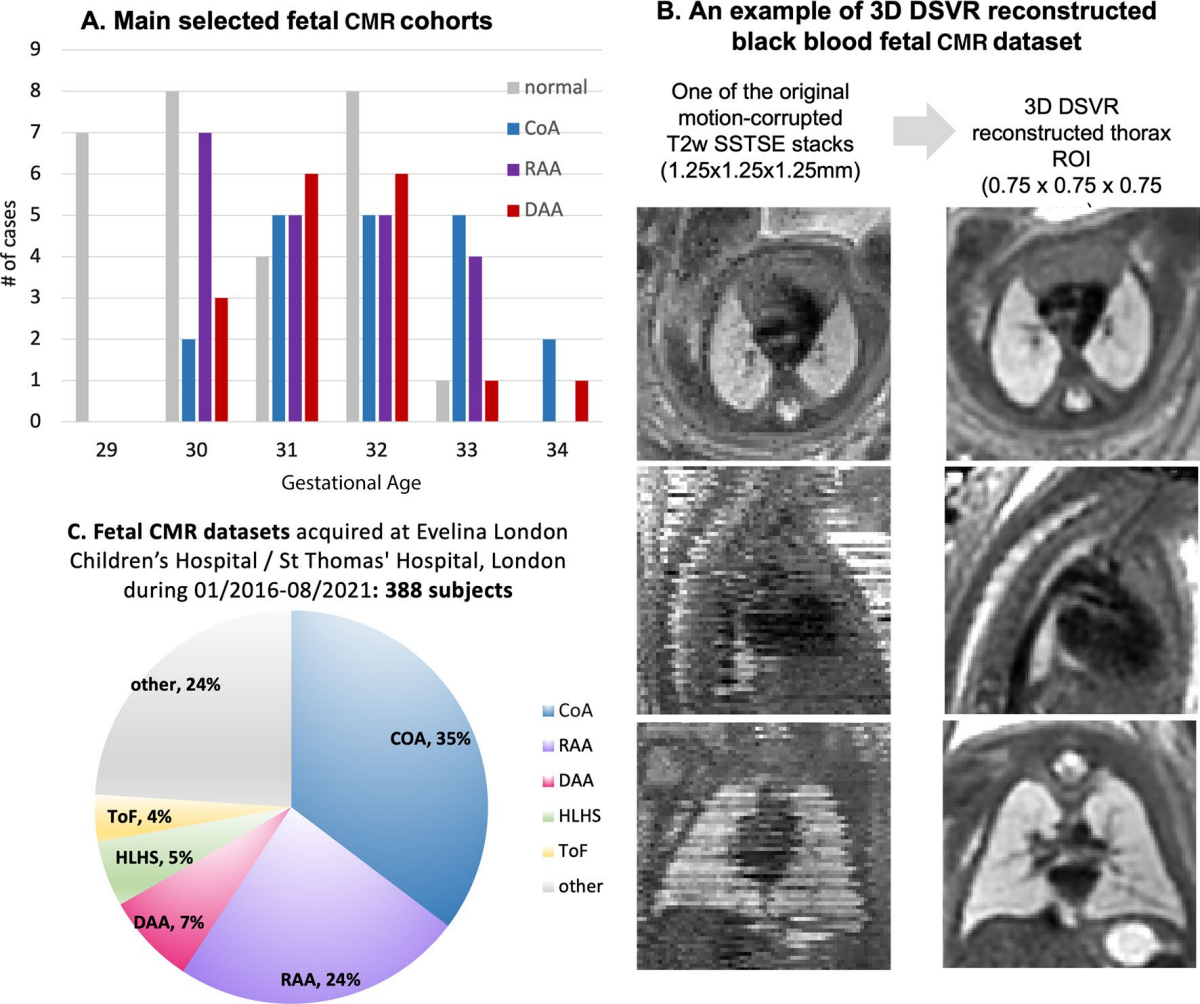
Investigated fetal cardiovascular magnetic resonance (CMR) cohorts (**A**) and an example of one of the original motion-corrupted stacks and the corresponding 3D DSVR reconstructed thorax for one of the black-blood T2-weighted SSTSE datasets (**B**). The entire fetal CMR cohort acquired at Evelina London Children’s Hospital during 2016-2021 is shown in (**C**). CoA, coarctation of aorta; DAA, double aortic arch; DSVR, deformable slice to volume registration; HLHS, hypoplastic left heart syndrome; RAA, right aortic arch, SSTSE, single shot turbo spine echo; T2w, T2 weighted; ToF, tetralogy of Fallot

As shown in Fig. 1C, suspected CoA ($35\%$), RAA ($24\%$) and DAA ($7\%$) represent the largest congenital heart disease (CHD) groups of the fetal CMR datasets acquired at Evelina London Children’s Hospital during 2016-2021 period. In terms of the image quality, on average, more than $\>90\%$ of all CMR cases had adequate image quality (3D reconstructions) acceptable for general diagnostic purposes (visibility of main vascular structures, e.g. Fig. 1B).

The main study cohort (86 subjects) (Fig. 1A) includes 28 healthy controls without reported cardiac, brain, body or placenta anomalies (’normal’), 20 cases with postnatally confirmed neonatal CoA and 38 vascular rings (21 RAA with aberrant left subclavian artery (ALSA), 17 DAA). We solely included high quality (high SNR and good visibility of all vascular structures) datasets of third-trimester singleton pregnancies (gestational age 29 to 34 weeks) without other major cardiovascular anomalies and anatomy variance (e.g., bilateral or left superior vena cava (SVC)) or significant extracardiac anatomical deviations.The numbers of the healthy controls, confirmed CoA and RAA cases were chosen to be approximately within the range of the number of the available good quality cases with isolated DAA (17), which was the smallest group. This case selection ensured the homogeneity of the cohort required for anomaly-specific atlas generation and the proof of concept evaluation of the feasibility of using multi-vessel segmentation of 3D fetal CMR (rather than a robust universal segmentation approach).

In addition, we also used randomly selected 3 early gestational age (normal anatomy; 22 weeks) and 3 low SNR (CoA, RAA, DAA; 29-32 weeks) datasets for assessment of the potential impact of various image quality factors on deep learning segmentation results.

Data acquisition was performed on a 1.5T CMR scanner (Ingenia, Philips Healthcare, Best, the Netherlands) using torso receiver array and T2w SSTSE: TR=15000ms, TE=80ms, voxel size 1.25x1.25x2.5mm, slice thickness 2.5mm and spacing 1.25mm with 9-11 stacks. No gating was used during T2 weighted (T2w) CMR acquisition. The 3D For the 3rd trimester datasets, the acquisition time varies between 1.5 - 3 minutes per stack depending on the ROI coverage[1]. The 3D static reconstructions of the fetal thorax (0.75mm isotropic resolution, standard radiological space) were generated using our novel automated deformable SVR (DSVR) [5] reconstruction pipeline [7]. 3D DSVR reconstruction provides spatial continuity of the vascular anatomy, which is essential for analysis of relative positioning of vessels in 3D. E.g., an example of the image quality before and after 3D DSVR image reconstruction is given in Fig. 1B.

### Generation of 3D heart atlases with vessel segmentation

**Fig. 2:**
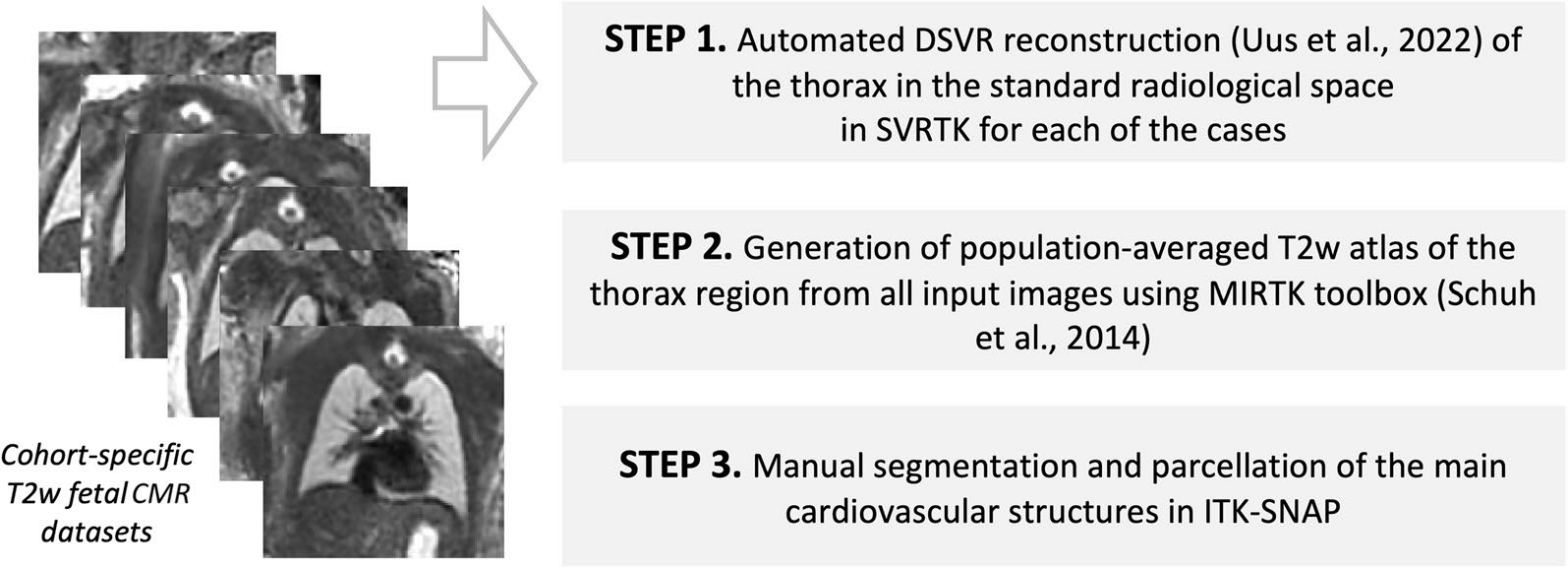
Main steps of the fetal heart atlas generation pipeline

The 3D atlases for each of the groups (normal, CoA, RAA+ALSA, DAA) were created using the classical Medical Image Registration Toolkit (MIRTK) atlas generation tool [18, 19] (Fig. 2). As preprocessing, the 3D reconstructed images from all cohort were affinely registered to the same standard space. The output atlases have 0.6mm isotropic resolution.

For each of the atlases, a clinician (MvP) with 5 years of fetal CMR experience manually segmented 20 labels of main cardiovascular structures using ITK-SNAP [20]. The atlases are publicly available online at SVRTK data repository [21].

### Automated segmentation of the fetal cardiac vessels

**Fig. 3:**
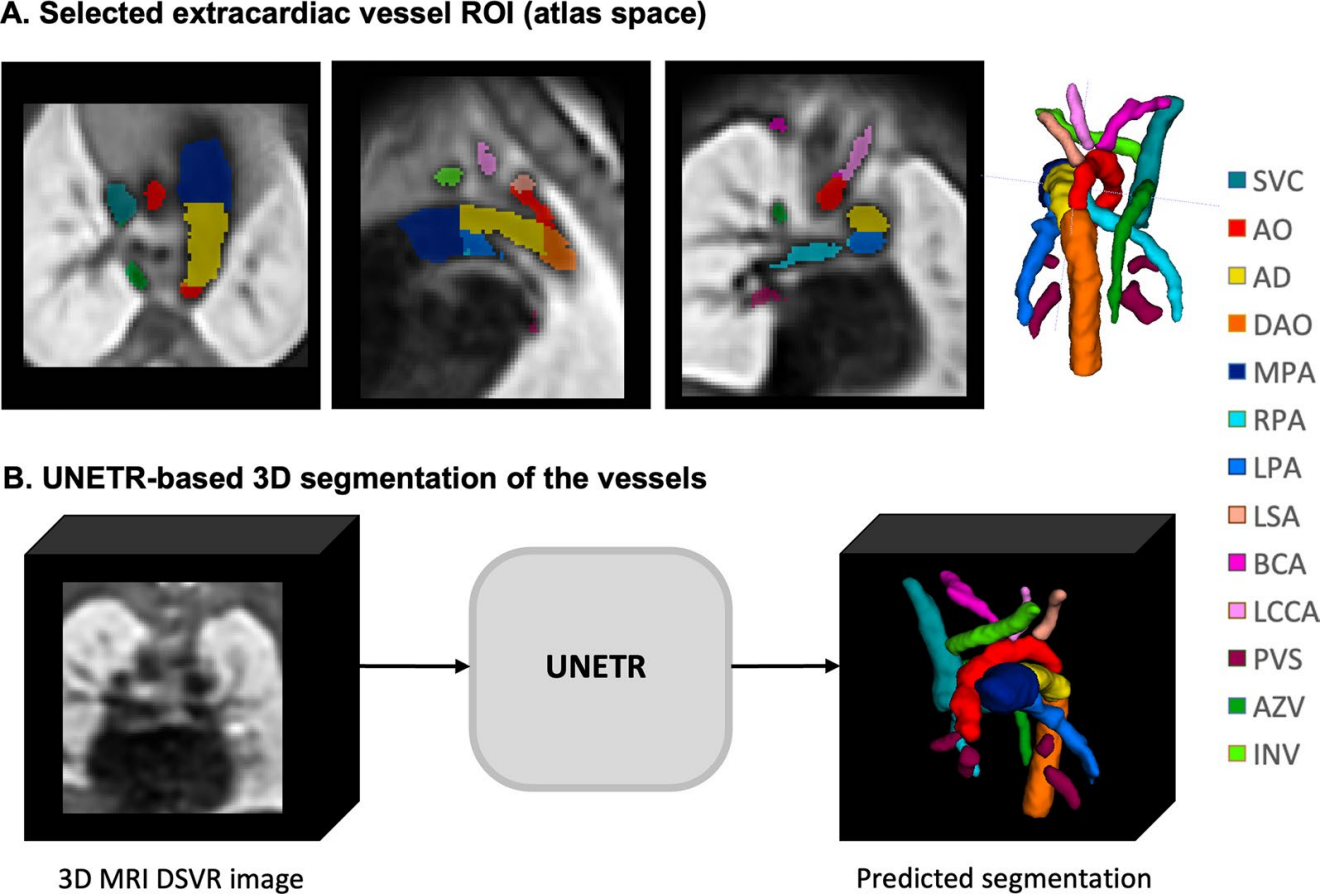
Definition of the vessels in the extracardiac region (**A**) and the proposed UNETR-based segmentation pipeline (**B**). AD, arterial duct; Ao, aorta; AZV - azygous vein; BCA, bracheocephalic artery; DAO, descending aorta; LCCA, left common carotid artery; LPA, left pulmonary artery; LSA, left subclavian artery; MPA, main pulmonary artery, PVS, pulmonary veins; RPA, right pulmonary artery

*Automated segmentation protocol:* For the automated segmentation protocol we selected 14 cardiac vessels, shown in Fig. 3A. These vessels are of primary interest in fetal CMR-based diagnosis of cardiac vascular anomalies, while black blood SSTSE sequences have limited value for visualisation of cardiac chambers.

*Deep learning model for automated segmentation:* We selected a vision transformer based deep neural network segmentation technique (UNETR) [22] to perform the automated multi-label 3D segmentation of the fetal vasculature in 3D DSVR CMR images (Fig. 3B), as it has shown to perform well for multi-label segmentation. The proposed segmentation pipeline was implemented in MONAI [23], and open-source Pytorch framework for medical artificial intelligence.

*Training datasets:* We used images from CoA, RAA and DAA cohorts including: 42 cases for training (14 from each cohort), 3 for validation and 6 for testing. The selection criteria were the isolated specific anomaly, absence of anatomical variations and the clear visibility and continuity of all vascular structures. The preprocessing included cropping to the extracardiac vessel region (e.g., Fig. 3A), affine registration to the atlas space and resampling with padding to 128x128x128 grid.

*Generation of labels for training:* The labels (14 different vessel regions, Fig. 3A) were generated using label propagation (LP) from cohort-specific atlases using deformable registration implemented in MIRTK software package [18]. This was followed by manual refinement of individual structures in all segmentations using ITK-SNAP [20], primarily for the aortic arch including head and neck vessels and azygous vein due to limited visibility (smaller size of structures) and partial volume effect. The RAA and DAA cases required more refinement than CoA case due to lower topological consistency. All labels were visually assessed and confirmed to be qualitatively acceptable.

*Training of the segmentation model:* The training was performed for 50000 iterations with the standard MONAI-based augmentation (random bias field, contrast adjustment, Gaussian noise and affine rotations $\pm 45^{{\circ }}$).

*Evaluation:* The performance was tested on 2 CoA, 2 RAA and 2 DAA cases qualitatively in terms of the vessel detection status (visual assessment: correct=100$\%$, partial=50$\%$, failed=0$\%$), and quantitatively by comparison to manually refined propagated labels in terms of recall, precision and Dice.

## Results

### 3D heart atlases

**Fig. 4:**
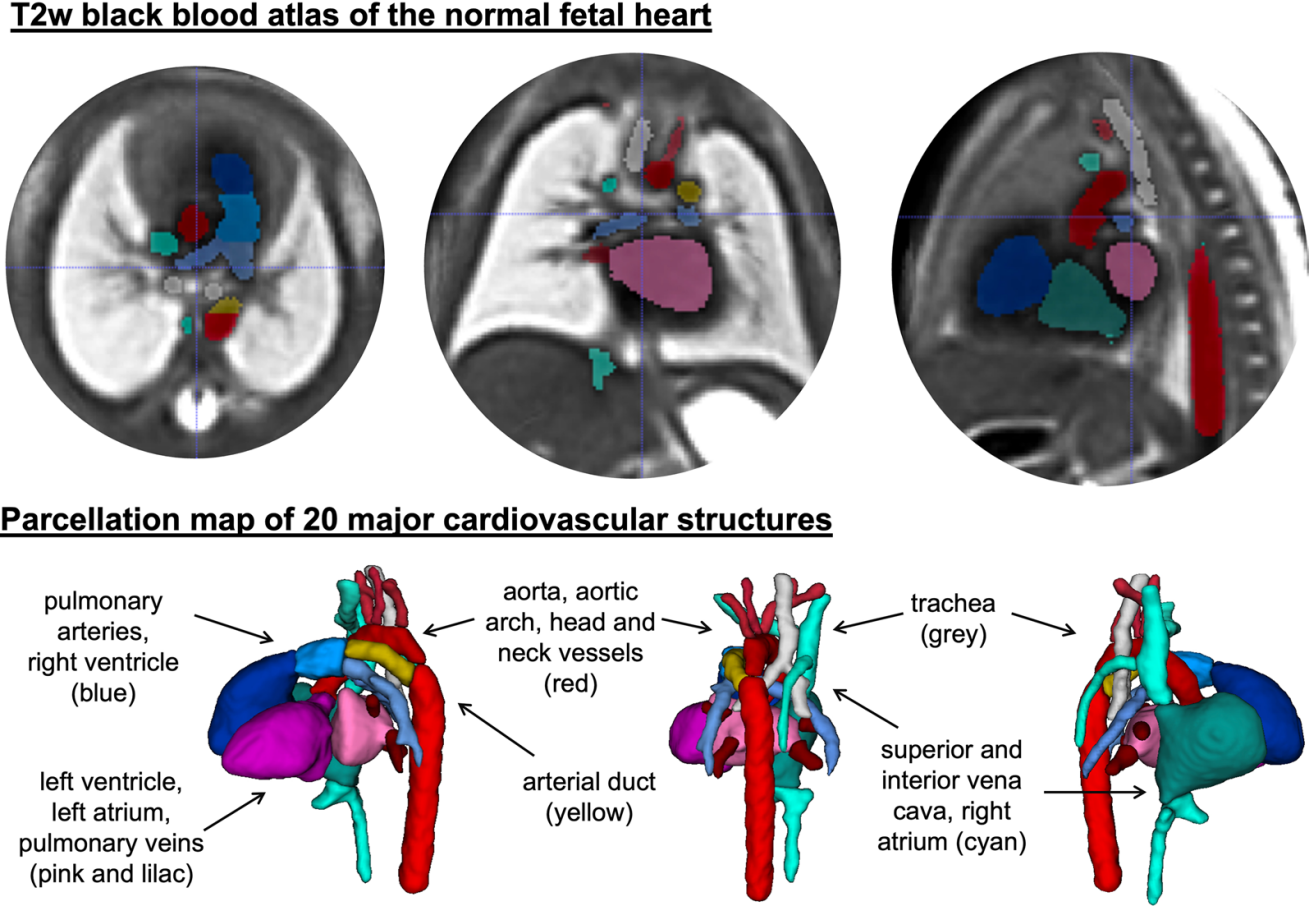
Generated atlas of the normal heart anatomy with the corresponding multi-label parcellation map

**Fig. 5:**
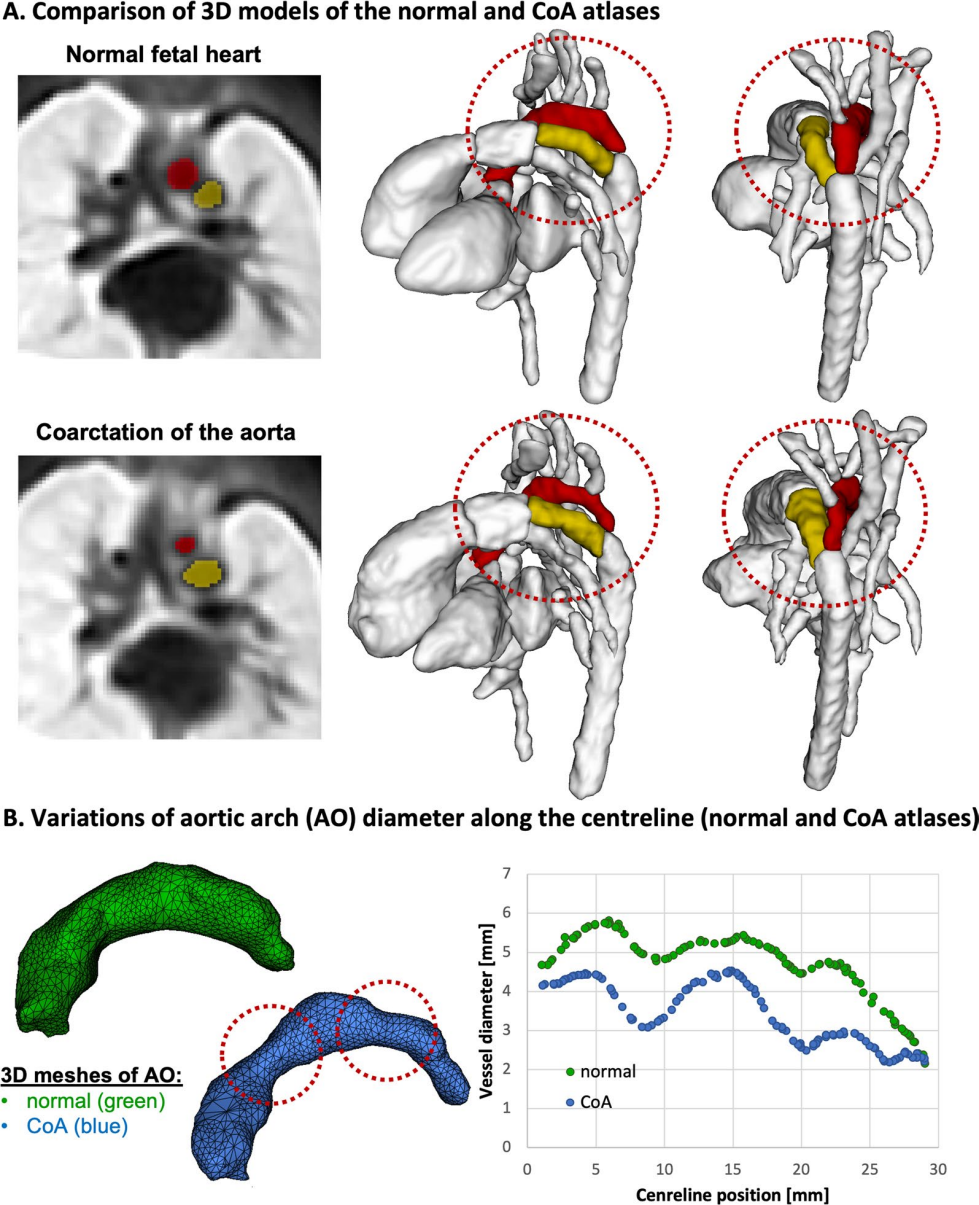
Generated 3D atlases of normal anatomy and averaged fetal arch anatomy as seen in postnatally confirmed CoA. (**A**) The 3D models show narrowing of the aortic arch (red) in the CoA atlas. The arterial duct is visualised in yellow and the rest the anatomy is set to white. (**B**) Comparison of the diameter changes along the aortic arch centreline between the normal (green) and CoA (blue) atlases

**Fig. 6:**
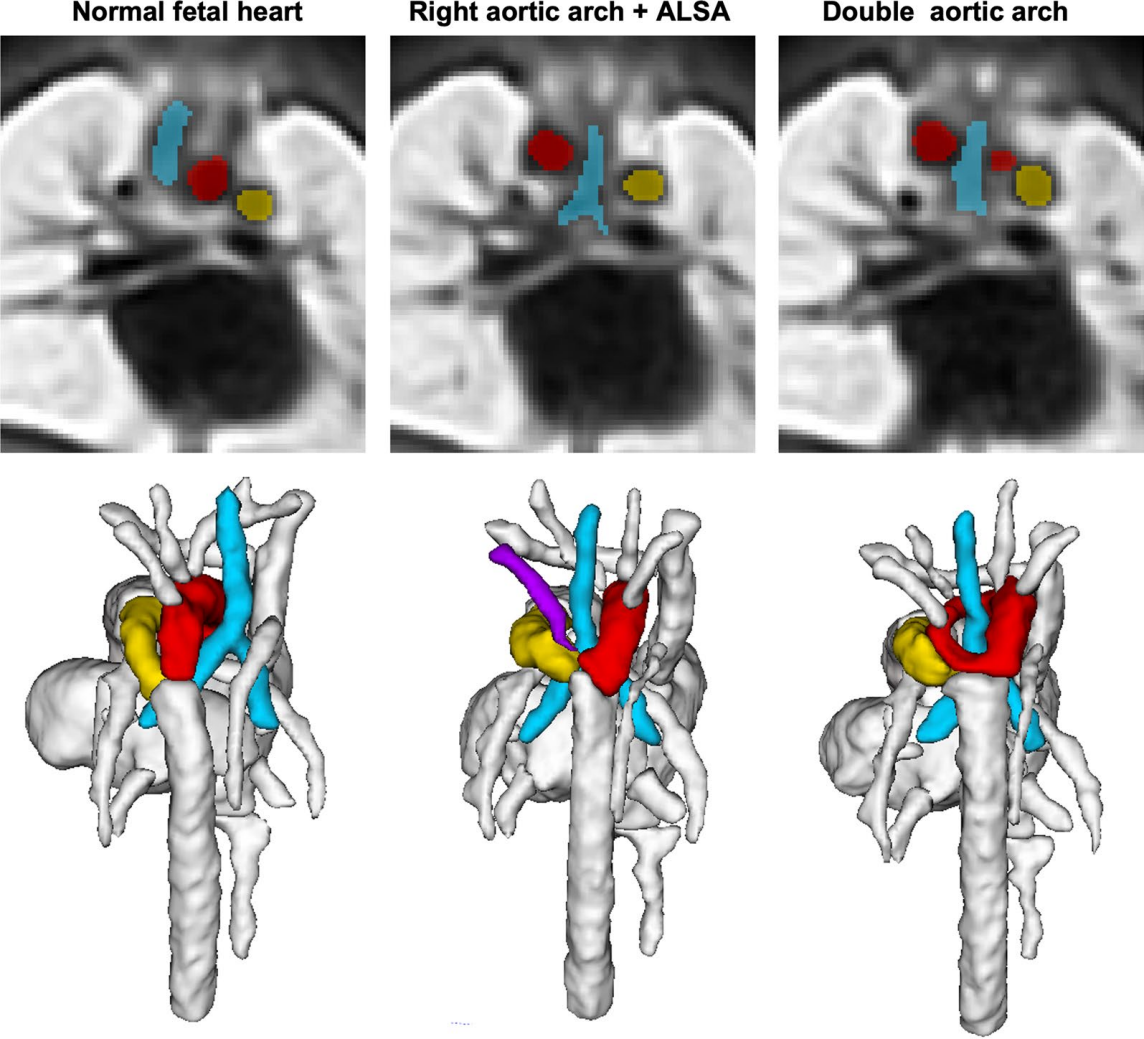
Comparison between the normal and abnormal RAA and DAA anatomy in the generated 3D atlases: differences in the relative position of the trachea (light blue) and aortic arch (red). The arterial duct is visualised in yellow and the aberrant left subclavian artery (ALSA) is visualised in lilac

Figs. 4, 5 and 6 show the generated 3D T2w atlases along with the corresponding multi-label parcellation maps. The anatomical accuracy of the atlases and the segmented structures were confirmed by a clinician (MvP) with 5 years of fetal CMR experience. Visual assessment of the atlases (e.g., Fig. 4A) demonstrates high contrast of the vessels as well as structural continuity of the blood pool. As expected, image quality of the atlases is higher then in individual cases.

The parcellation maps (e.g., Fig. 4B) include 14 major cardio vascular structures (including aorta and head and neck vessels (red), arterial duct (yellow), inferior and SVC and innominate vein (cyan), pulmonary arteries (light blue) and pulmonary veins (lilac). Additional maps are the four heart chambers, the trachea is shown in grey.

The back blood T2w SSTSE sequence is primarily used for visualisation of vessels and has limited value for inspection of cardiac chambers in individual subject studies due to low contrast. However, averaging of the signal from multiple aligned 3D images during the atlas construction enhances the contrast and produces smooth outline of the chambers. The segmentations of the chambers were added to the atlases primarily for completeness.

The 3D model of the CoA atlas shown in Fig. 5A has a pronounced narrowing of the aortic arch in comparison to the normal anatomy. This is reflected in the narrower diameter along the centreline (Fig. 5B). The diameter was measured using VMTK centreline extraction technique [24]. Of note, the CoA atlas represents an average of all CoA input cases which displayed variation in degree of great artery asymmetry and isthmus position as seen previously [6].

The RAA and DAA atlases, Fig. 6 clearly demonstrate the expected differences in the relative position of the aortic arch(es) and aortic branching to the trachea when compared to the normal anatomy of a single left aortic arch [25]. The ALSA is highlighted in lilac in the RAA atlas.

### Automated cardiac vessel segmentation

**Fig. 7:**
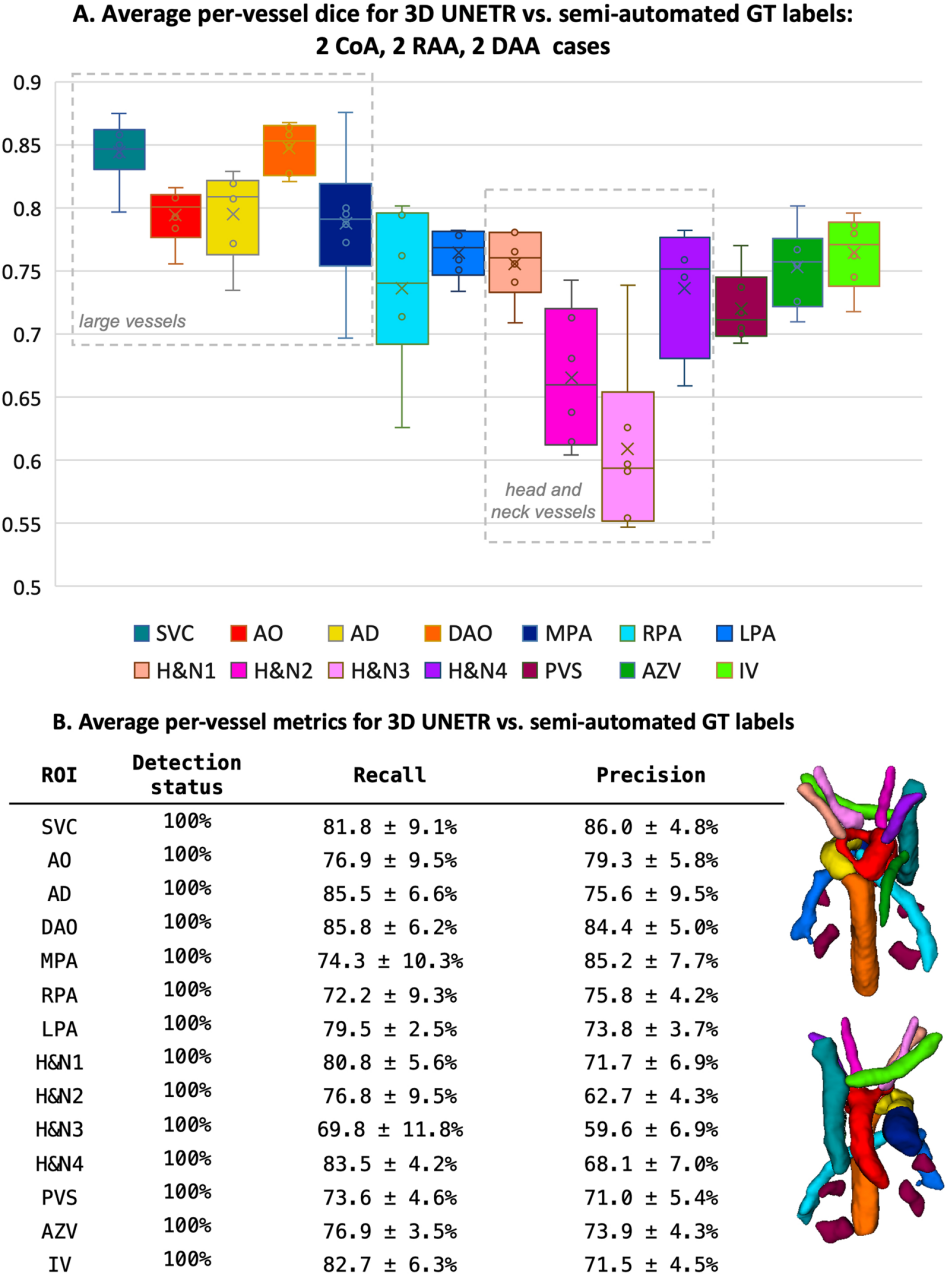
Evaluation of 3D UNETR segmentation for 14 extracardiac vessel ROIs vs. the ground truth (GT) manually refined atlas propagated labels: including average Dice (**A**), precision, recall and detection detection status (**B**)

*Evaluation of UNETR segmentation:* The results of testing of the trained multi-label 3D UNETR segmentation network on 6 CoA, RAA and DAA cases are summarised in Fig. 7. The UNETR correctly detected all vessels in all test subjects (100$\%$) with different anomalies. This is confirmed by relatively high Dice coefficients for all structures (around 0.8 for larger vessels and around 0.75 for most of the smaller vessels) in agreement with the adequate recall and precision.

Note that for some head and neck vessels Dice coefficients are lower (0.55-0.7) due to their smaller sizes. Additionally, even after manual refinement, the reference ground truth labels are prone to inconsistencies due to the uncertainties stemming from the partial volume effect and varying per-vessel intensity contrast levels, which further contributes to lower Dice scores.

Nonetheless, these results confirm the feasibility of using deep learning models for multi-label segmentation for small vascular structures in a mixture of abnormal datasets with different anatomy.

In addition, Table  1 shows the quantitative comparison of the fine-edited label propagation (GT) and UNETR outputs with manual segmentations that were performed similarly to the original protocol in [1] (based on intensity thresholding followed by manual refinement). Since there are no specified fixed cutoff levels for segmenting individual vessels or bifurcations or the signal intensity corresponding to the lumen boundaries, the Dice values of comparison with the manual segmentations are expected to vary. However, the relatively acceptable Dice results confirm the feasibility of using both of the proposed approaches.

**Table 1:** Comparison between the manual labels (thresholding $+$ manual refinement), the ground truth (GT) manually fine-edited atlas propagated labels and UNETR outputs: average Dice per vessel ROI

Method	SVC	AO	AD	DAO	MPA	RPA	LPA
LP+editing	$0.77\pm 0.03$	$0.68\pm 0.04$	$0.73\pm 0.03$	$0.77\pm 0.4$	$0.78\pm 0.05$	$0.64\pm 0.01$	$0.57\pm 0.05$
UNETR	$0.76\pm 0.05$	$0.68\pm 0.04$	$0.73\pm 0.03$	$0.78\pm 0.04$	$0.73\pm 0.06$	$0.66\pm 0.05$	$0.57\pm 0.07$
Method	INV	PVS	LSA	BCA	LCCA	AZV	ALSA
LP+editing	$0.68\pm 0.02$	$0.48\pm 0.08$	$0.68\pm 0.05$	$0.58\pm 0.05$	$0.47\pm 0.23$	$0.64\pm 0.06$	$0.68\pm 0.05$
UNETR	$0.72\pm 0.06$	$0.48\pm 0.13$	$0.71\pm 0.07$	$0.66\pm 0.09$	$0.58\pm 0.19$	$0.70\pm 0.02$	$0.76\pm 0.05$

**Fig. 8:**
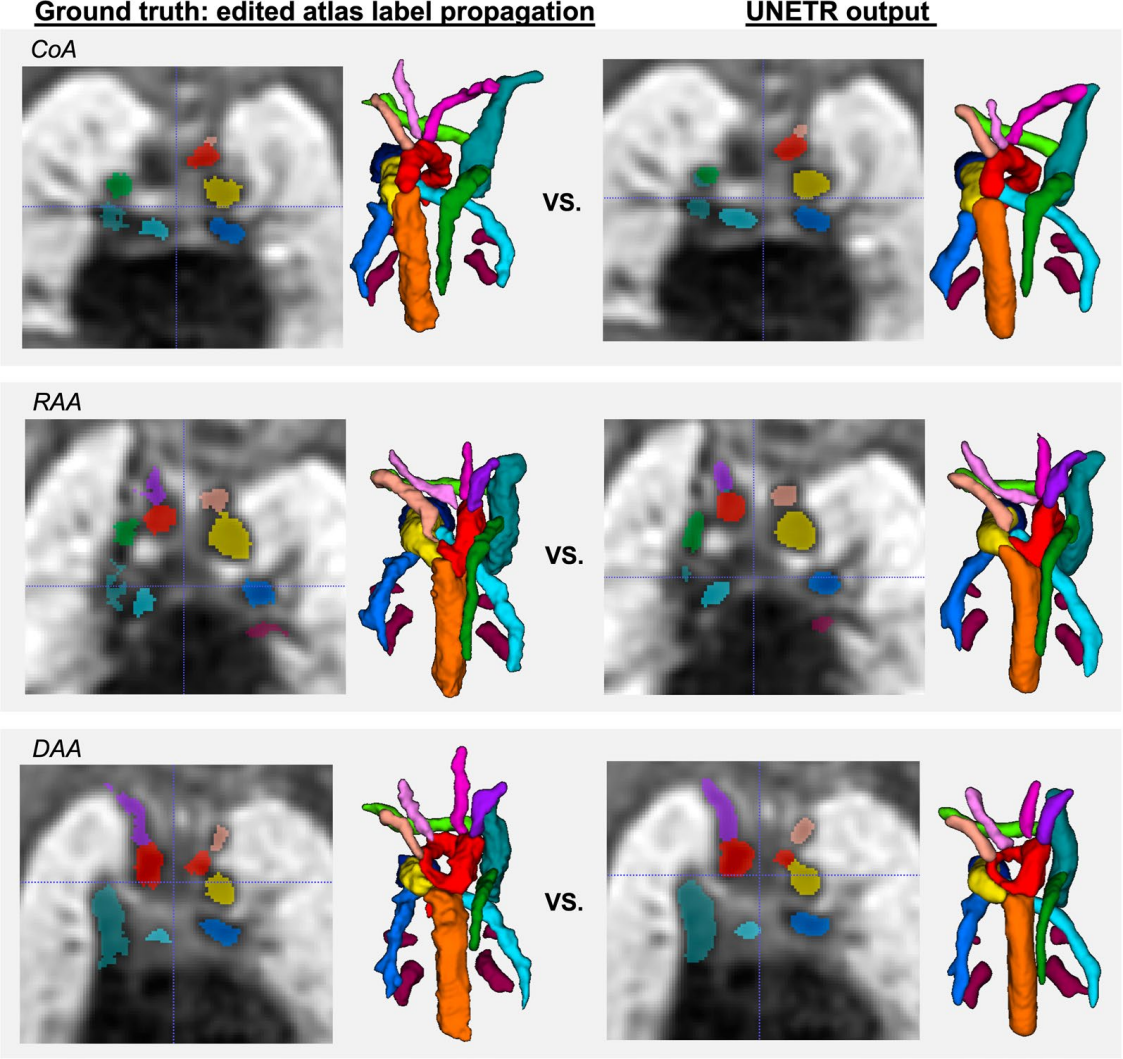
Examples of comparison between the manually refined propagated labels (GT) and UNETR outputs for three different aortic arch anomaly groups

*Visual comparison:* The examples of visual comparison of the ground truth manually refined labels propagated from the atlases vs. UNETR outputs for different anomaly groups are shown in Fig. 8. While label propagation provided sufficiently good localisation of the vessels, there are minor inconsistencies and patchy appearance of the labels at the vessel boundaries. This is primarily related to the expected limitations of intensity-based registration methods. These irregularities were partially resolved in the UNETR output, which produced significantly more realistic and smoother boundaries and potentially corrected minor errors.

**Fig. 9:**
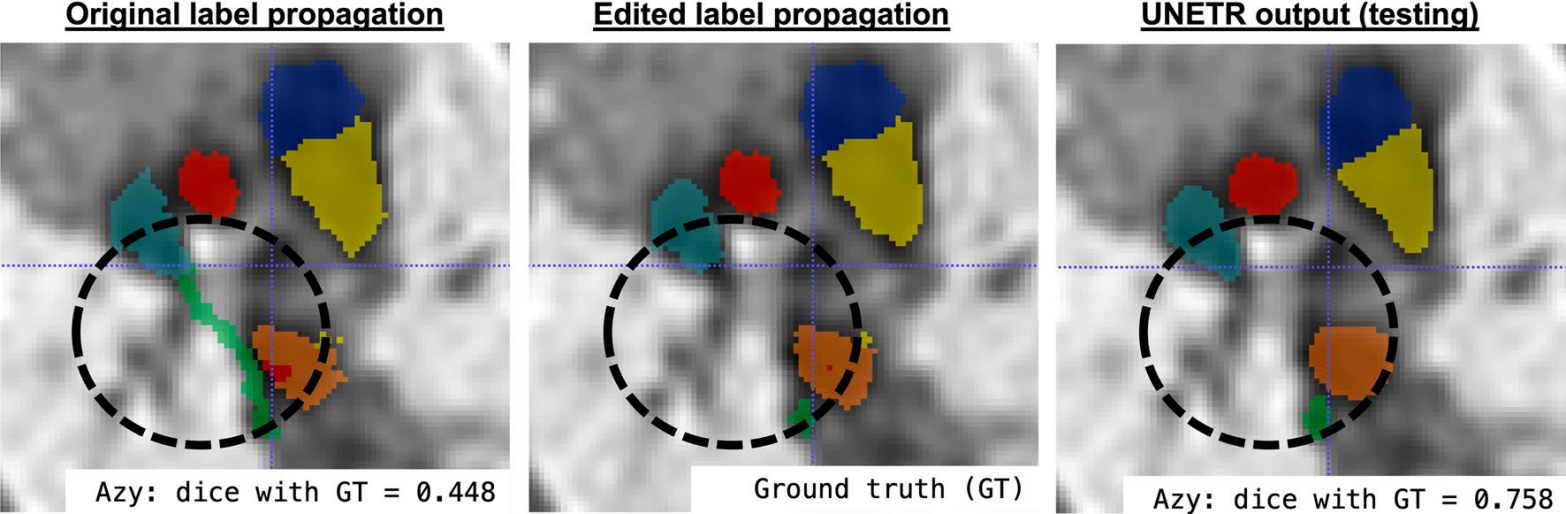
An example of label propagation error in one of the testing datasets that required manual refinement: incorrect azygous vein segmentation

*UNETR and label propagation:* Our results also confirm the feasibility of using labels propagated from average atlases for training of deep learning segmentation, which significantly reduces required dataset preparation time compared to manual-only segmentations. However, even without extreme anatomical deviations, label propagation outputs tend to require a certain amount of manual editing. Fig. 9 shows an example of failed label propagation segmentation of the azygous vein for one of the test datasets. It required manual correction while the UNETR produced correct segmentation. In total, for all datasets used in training and testing of the network, either minor or significant manual editing was required in approximately $30\%$ of all individual structures, correspondingly.

An additional experiment for investigation of the potential limitations of the network with respect to suboptimal image quality is available in Appendix. It includes the unseen types of fetal CMR datasets: 3 early gestational age (22 weeks) and 3 low SNR cases.

## Discussion

In general, the main aim of this work was focused on improvement of 3D CMR-based prenatal diagnosis for specific types of vascular CHD via formalisation of the diagnostic protocol that is currently at the early stage of integration into clinical practice [1] as well as optimisation of the current 3D vessel segmentation approach via automation (to reduce the time) and providing a potential solution to decrease inter-observer variability.

The proposed black-blood atlases demonstrate the group-averaged appearances of cardiovascular structures as seen on 3D fetal CMR in both normal and 3 subgroups of CHD. The correctness of the vascular anatomy in all atlases was confirmed by clinicians with fetal CMR experience. We also formalised the first protocol of parcellation of the major cardiovascular structures which provides the basis for development of automated segmentation methods.

In the context of prenatal diagnosis of CHD with fetal CMR, the main aim of segmentation is 3D visualisation of the cardiovascular structures to demonstrate the relative position and dimensions of the relevant vessels, for example in cases with abnormal arch anatomy. One of the main requirements to any segmentation-based image analysis is the consistency of segmentation protocols in terms of the inter- and intra-observer variability. Potentially, this can be achieved by applying automated segmentation methods.

An important application of the created atlases is label propagation for multi-vessel segmentation and it produces relatively consistent results for the normal or close-to-normal anatomy in CoA cases. However, DAA and RAA cases are subject to higher topological variations leading to limited segmentation quality. Varying image quality is another aspect that impedes expected segmentation quality in registration-based methods. The modern advanced deep learning-based segmentation methods provide an alternative operational solution but they also require large and high quality training datasets.

In this work, we utilised manually refined labels propagated from the atlases for UNETR training, which significantly reduced time for preparation of the datasets and provided additional consistency of the labels. The results of testing confirmed that a single deep neural network can be successfully used for multi-label segmentation of datasets with different types of abnormal aortic arch anatomy. In terms of the performed quantitative evaluation results, despite the 100% detection status, the lower dice for small vessels however smaller vessels would primarily require detection status due to the lower reliability, visibility and image quality.

The dataset selection criteria for this study were high SNR and only isolated anomalies without any significant anatomy deviations. This selection strategy was employed because this work focuses on the assessment of the general feasibility of using multi-vessel segmentation for 3D fetal CMR and formalisation of the parcellation protocol rather than a robust universal segmentation network.

These results confirm that automated deep learning methods can provide sufficiently accurate 3D segmentation of fetal anatomy in 3D CMR despite small size of vessels (vs. available resolution) and relatively small number of training datasets especially given heterogeneity in CHD groups. Automation of segmentation could significantly increase the efficiency by reducing laborious manual delineation and, in longer term, reduce inter-observer variability which is relevant for both visualisation and quantitative measurements (including vessel biometry and shape analysis). The fact that the network could differentiate between the different aortic arch anomaly anatomies also indicates the feasibility of using deep leaning for prediction of the vascular anomaly type directly from 3D CMR images.

One the major advantages of using neural networks vs. classical atlas-based segmentation approaches is that atlas-based segmentation requires specific atlases for specific anomalies (e.g., RAA atlas cannot be used for other anomalies) while we demonstrated that a single neural network can be used for three different AA anomaly cases. Using larger training datasets with higher anatomical variability (e.g., bilateral SVC) and varying image quality (e.g., early gestational age, low SNR) would allow implementation of a more general solution.

In summary, in future, this proof of concept work could potentially lead to a wider application for 3D reconstructed fetal CMR for diagnosis of CHD as well as providing the basis for development of novel methodologies (e.g., advanced deep learning automated segmentation and diagnosis). This will also provide a possibility to review bigger cohorts without inter-observer variability.

### Limitations

The reliability of segmentation results highly depends on the degree of anatomical variability and presence of other anomalies in the training datasets. While the proposed solution provides a proof of concept it was based on the subset of cases with isolated aortic arch anomalies and good image quality. Implementation of a universal solution that could be used for routine clinical scans would require preparation of a significantly larger training dataset. In addition to the wider range of heart anomalies, it should include datasets from different CMR acquisition protocols, different gestational age ranges and with varying image quality. This will also require a large-scale evaluation of the visualisation of individual anatomical structures and measurements. It would also be beneficial to add bright blood sequence atlases for cardiac chambers in order to extend the definition of the normal and abnormal appearance of the fetal heart anatomy and provide the basis for the multi-channel whole heart segmentation pipeline.

## Conclusions

This work introduced the first 3D black-blood fetal CMR atlases of normal fetal cardiovascular anatomy and three types of abnormal arch anatomy (CoA, RAA with ALSA and DAA) along with detailed parcellation maps of the major cardiovascular structures. This is a first step towards automation of the segmentation pipeline of 3D motion-corrected CMR [1]. We demonstrated the feasibility of using deep learning for automated multi-label vessel segmentation for different types of isolated aortic arch arch anomalies.

Future work will need to focus on optimisation of the deep learning segmentation pipeline for a wider range of fetal CHD abnormalities and anatomical variations as well as different acquisition protocols and automated vessel biometry.

### Supplementary Information

**Additional file 1: Fig. S10** Examples of assessment of segmentation performance on 3 low SNR (COA, RAA, DAA) and 3 early GA (normal anatomy, 22 weeks) cases

## Data Availability

The generated 3D fetal heart MRI atlases with parcellation maps are publicly available at the online SVRTK data repository [21].

## Appendix

In order to investigate the potential limitations of the label propagation and UNETR approaches in terms of the general robustness to image quality and variance in appearance of vascular structures (suboptimal datasets), we performed segmentation on previously unseen fetal CMR data types (not presented in the training dataset). This included 3 low SNR (CoA, RAA, DAA; 29-32 weeks) and 3 early GA (normal anatomy; 22 weeks) cases. Both of these groups pose a challenge to automated segmentation due to the limited visibility of vascular structures. E.g., in early GA cases, it is difficult to distinguish finer vessels (due to small size vs. image resolution) because of the significantly decreased image contrast and visibility. Low SNR leads to the presence of gaps in structural continuity of the individual vessels.

The segmentation results were assessed qualitatively in terms of the vessel detection / segmentation quality status. The results presented in Additional file 1: Fig. S10 show that both methods provided relatively similar good performance for the large vessels (e.g., aorta and vena cava) with 100$\%$ grades for the majority of cases (detected vessel without under- or over-segmentation). However, the deep learning approach produced only partial segmentation for a large proportion of finer vessels due to its sensitivity to signal contrast and because this type of data was not used during the training. This could be potentially resolved by extending the training datasets or incorporating deep learning registration into the pipeline.

Low image quality also notably affects registration accuracy for label propagation resulting in over- (or under-) segmentation that requires additional manual editing. At the same time, in general, the limited visibility of structures reduces the reliability of any segmentation method (even manual) in terms of the validity of output 3D models that cannot be fully verified.

## Abbreviations

AD: Arterial duct
AO: Aorta
ALSA: Aberrant left subclavian artery
AZV: Azygos vein
BCA: Brachiocephalic artery
CoA: Coarctation of the aorta
CHD: Congenital heart disease
CMR: Cardiovascular magnetic resonance g
DAA: Double aortic arch
DAO: Descending aorta
DSVR: Deformable slice-to-volume registration
GA: Gestational age
IV: Innominate vein
IVC: Inferior vena cava
LA: Left atrium
LCCA: Left common carotid artery
LPA: Left pulmonary artery
LSA: Left subclavian artery
LV: Left ventricle
MPA: Main pulmonary artery
MRI: Magnetic resonance imaging
PVS: Pulmonary veins
RA: Right artrium
RAA: Right aortic arch
RCCA: Right common carotid artery
RPA: Right pulmonary artery
RSA: Right subclavian artery
RV: Right ventricle
SNR: Signal-to-noise ratio
SSTSE: Single shot turbo spin echo
SVC: Superior vena cava
SVR: Slice-to-volume registration
UNETR: UNEt TRansformers
