# Supplementary figures and images for: 3D black blood cardiovascular magnetic resonance atlases of congenital aortic arch anomalies and the normal fetal heart: application to automated multi-label segmentation
Source: J Cardiovasc Magn Reson. 2022 Dec 15;24:71. doi: 10.1186/s12968-022-00902-z (PMC9753334; doi:10.1186/s12968-022-00902-z)

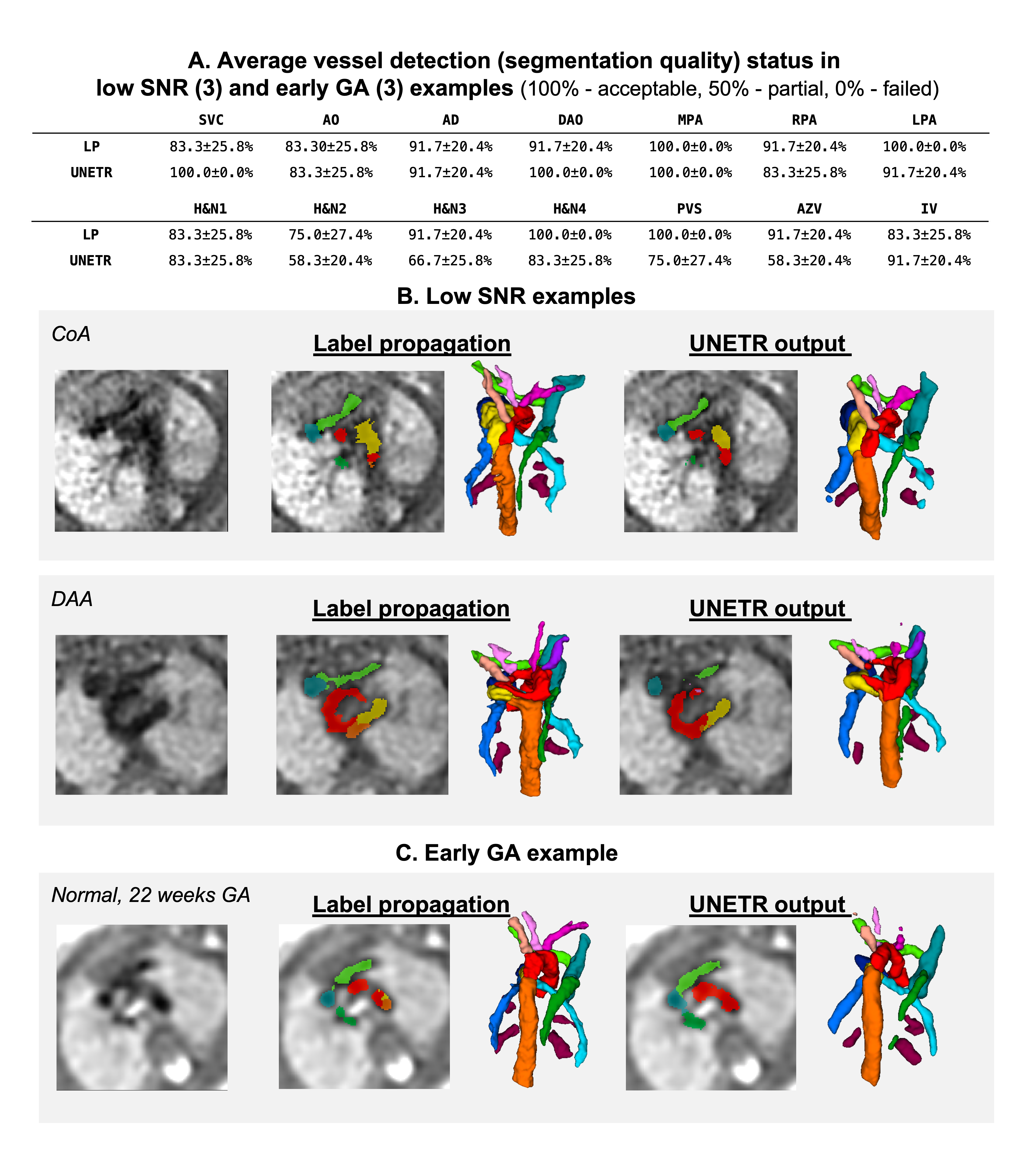

Supplement: Supplementary file 1 — Additional file 1: Fig. S10 Examples of assessment of segmentation performance on 3 low SNR (COA, RAA, DAA) and 3 early GA (normal anatomy, 22 weeks) cases [file 12968_2022_902_MOESM1_ESM.png]
